# Role of regulatory T cells in spinal cord injury

**DOI:** 10.1186/s40001-023-01122-6

**Published:** 2023-05-09

**Authors:** Hao Chen, Hao Peng, Ping-Chuan Wang, Tao Zou, Xin-min Feng, Bo-wen Wan

**Affiliations:** Northern Jiangsu People’s Hospital Affiliated to Yangzhou University/Clinical Medical College, Yangzhou University, Yangzhou, 225000 China

**Keywords:** Spinal cord injury, Regulatory T cells, Neuroinflammation

## Abstract

Spinal cord injury is an intricate process involving a series of multi-temporal and multi-component pathological events, among which inflammatory response is the core. Thus, it is crucial to find a way to prevent the damaging effects of the inflammatory response. The research has found that Treg cells can suppress the activation, proliferation, and effector functions of many parenchymal cells by multiple mechanisms. This review discusses how Treg cells regulate the inflammatory cells to promote spinal cord recovery. These parenchymal cells include macrophages/microglia, oligodendrocytes, astrocytes, and others. In addition, we discuss the adverse role of Treg cells, the status of treatment, and the prospects of cell-based therapies after spinal cord injury. In conclusion, this review provides an overview of the regulatory role of Treg cells in spinal cord injury. We hope to offer new insights into the treatment of spinal cord injury.

## Background

Spinal cord injury (SCI) is a devastating neurological state that may result in motor, sensory, and autonomic dysfunction [1]. Its prevalence has risen from 236 to 1298 cases per million people globally over the past 30 years. According to estimates, there are between 250,000 and 500,000 SCI cases yearly [2]. However, due to the disease’s complex pathophysiological mechanisms and technical limitations, treatment options are limited [3, 4]. Therefore, developing a multifaceted and multidisciplinary SCI treatment is a critical unmet need.

SCI is divided into primary and secondary injury, according to its pathological process. The primary injury has no predictable or effective treatment. At present, research at home and abroad has focused on the secondary injury. The secondary injury is divided into three stages according to the time after injury in humans [5, 6]: the acute stage (2 days after injury), the subacute phase (2–14 days after injury), and the chronic phase (14 days to 6 months). SCI is a dynamic process, and multiple factors determine its progression and prognosis. Microenvironment imbalance and the infiltration of parenchymal cells are key to secondary spinal cord injury [7]. For example, innate parenchymal cells in the central nervous system respond rapidly after SCI, including microglia, astrocytes, and oligodendrocyte progenitor cells. Those cells express pro-inflammatory factors, and recruit and activate the peripheral immune cell. This inflammatory response may persist for days or weeks, leading to myelin degeneration, neuronal apoptosis, and scarring, thereby worsening neurological dysfunction [8, 9]. Thus, it is crucial to find a way to prevent the damaging effects of the inflammatory response.

**Fig. 1:**
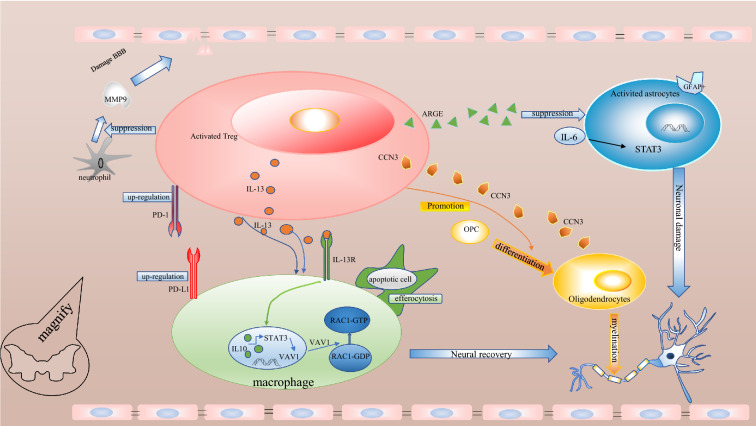
The regulation of Treg cells in SCI. These cells include macrophages/microglia, oligodendrocytes astrocytes, and others

Regulatory T cells (Treg cells) are a small subset of CD4 + T cells that are identified by the expression of several hallmark proteins, including CD25, Foxp3, and Helios. They are essential for maintaining immunological homeostasis and dominant self-tolerance, and their dysfunction (for instance, owing to Foxp3 gene mutation) results in a fatal autoimmune disease and immunopathology [10]. Treg cells can inhibit the activation, proliferation, and effector functions of other immune cells [11]. Multiple mechanisms are involved in Treg-mediated suppression, including cell contact-dependent and humoral factor-mediated ones, such as cell surface molecules (CTLA-4, CD25, TIGIT, CD39, and CD73), cytokines (IL-2, IL-10, TGF-β, and IL-35), and secreted or intracellular molecules (granzyme, cyclic AMP, and IDO) [12, 13]. As excessive neuroinflammation can amplify SCI pathologies, the immunosuppressive properties of Treg cells are expected to mitigate the impact of SCI [14]. This article summarizes the latest research on Treg cells in SCI (Fig. 1).

### Treg cells repair the spinal cord by regulating macrophages/microglia

As the primary innate immune cells, the roles of microglia and peripherally derived macrophages after SCI have attracted much attention in recent years. Immediately after SCI, microglia are activated, and the cytokines and chemokines they secrete lead to the recruitment of neutrophils and macrophages. The first wave of macrophages starts approximately 3 days after injury, peaks at 7 days, and endures for a long time [15]. Although macrophages may develop a microglia-like morphology, they recruited in the brain parenchyma are not match the host microglia's transcriptional signature [16, 17]. Due to the phenotypic and antigenic similarities between microglia and macrophages after SCI, it is difficult to differentiate them, so we collectively refer to them as macrophages/microglia. It is now abundantly clear that macrophages/microglia play a central role after SCI [14]. Many studies have shown that Treg cells have a regulatory effect on macrophages/microglia.

First, Treg cells can repair the spinal cord by affecting the polarization of macrophages/microglia. It has been reported that microglia/macrophages are highly plastic, and their phenotypes depend on different microenvironmental conditions [18]. M1-type or neurotoxic microglia secreting many pro-inflammatory factors (TNF-α, IL-1β, and IL-6) are not unfavourable to tissue repair. M2-type or neuroprotective microglia secreting inflammatory factors (IL-4 and IL-10) promote the recovery of nerve function [19, 20]. Single-cell and high-plex-omics techniques accurately more characterize phenotypes [21], but the concept of an activation spectrum based on pro- vs. anti-inflammatory characteristics remains a helpful framework. Paolicelli et al. brought together a team of multidisciplinary experts to address these problems. Unfortunately, the profession has not yet agreed on some nomenclature issues [22].

Liu et al*.* found that inflammation-related genes, such as ApoD, and downstream cytokines IL-6 and TNF- were elevated in microglia in Treg-depleted animals after SCI. TNF- and IL-6 participate in canonical STAT3 activation signalling, inhibiting function recovery in various neuroinflammatory disorders. Eventually, Treg depletion caused microglia to develop a pro-inflammatory phenotype [23]. Treg cells can also express programmed cell death protein 1 (PD-1) to influence the polarization of macrophages/microglia. The major of research indicates that the PD-1/PD-L1 signalling pathway sustains or promotes Treg cell expansion, Foxp3 expression, and immunosuppressive function [24–26]. PD-1 inhibits the polarization of macrophages/microglia towards the pro-inflammatory type post-SCI [27]. He et al. [28] observed PD-1 upregulation in infiltrating Treg cells and PD-L1 upregulation on post-SCI macrophages/microglia through flow cytometry. The results of the co-culture study showed that PD-1 maintained the expression of Foxp3, IL-10, and TGF-β after Treg cells contact with pro-inflammatory macrophage/microglia. The above experiment suggested that PD-1 secreted by Treg cells is essential for Treg cells to maintain their identity and anti-inflammatory activity. In addition, Yang et al*.* [29] found that Treg cells inhibited microglia activation and restricted TNF-α, IL-1β, and MMP-2(Pro-inflammatory factors) expression of microglia through the JNK/ERK pathway and NF-κB. Shi et al. [32] found that the osteopontin (OPN)–integrin receptor axis plays a part in the communication between Treg cells and microglia. OPN has significant roles in immune modulation and is expressed by peripheral Foxp3 + Treg cells, according to earlier studies [33]. They found that OPN, a protein produced by Treg cells, functions mechanistically by increasing microglial reparative activity and oligodendrogenesis by binding to integrin receptors on microglia. Subsequently, increasing the number of Treg cells by administering IL-2:IL-2 antibody complexes enhanced the integrity of the white matter and restored neurological functions over time.

Previous studies have shown that M1-like are mixed with M2-like types within 7 days after SCI; however, the proportion of M2-types gradually decreases to disappears, while M1-types gradually increase and dominate for a long time [15]. It should be noted that M2-type secrete cytokines, such as transforming growth factor-β (TGF-β), platelet-derived growth factor (PDGF), can promote the formation of fibrous scarring and are not favourable to SCI recovery [30]. Therefore, the pro-inflammatory/pro-reparative balance is essential for immune homeostasis after SCI.

Second, research uncovered that Treg cells could repair the spinal cord by enhancing efferocytosis by macrophage/microglia. Treg cells are poised to play an essential role in inflammatory resolution by suppressing the inflammatory activity of innate and adaptive immune cells and secreting substances that promote tissue repair [31]. Proto et al. [32] established an acute zymosan-induced peritonitis inflammation model to test the theory that Treg cells promote efferocytosis during inflammation resolution. They found that Treg cells promote the efferocytosis of macrophages, a critical effector arm of inflammation resolution. They further found that IL-13 produced by Treg cells promotes macrophages/microglia to release IL-10, which acts in an autocrine-paracrine manner to stimulate apoptotic cell engulfment through a Vav1–Rac1–STAT3-mediated mechanism. These data indicated that Treg cells are crucial for the clearance of apoptotic cells during the resolution phase. The above experimental conjecture has not been confirmed in the spinal cord, but it provides new insights and ideas for our study of Treg cells on SCI.

Finally, Treg cells can repair the spinal cord by affecting the pyroptosis of macrophages/microglia. Pyroptosis is a pro-inflammatory form of programmed cell death that is an uncontrollable inflammatory damage brought on by the organism’s overzealous response to external stimuli. It is closely correlated with the degree of oxidative stress, immune response, and another intracellular environmental homeostasis [32, 33]. Inflammasomes are activated by intracellular danger signals after injury, resulting in cell swelling, rupture, and the release of inflammatory mediators [34]. An inflammatory cascade is caused by the released inflammatory mediators, which attract in more immune cells [35]. Hence, avoiding pyroptosis following SCI can decrease the severity of the secondary inflammatory injury and enhance the patient's prognosis [36]. Xiong et al*.* discovered that exosomes produced by Treg cells improve functional recovery. Their studies confirmed that Treg cells used exosomal miR-709 to target the NKAP to decrease microglia pyroptosis and promote motor function recovery following SCI [16].

### Treg cells repair the spinal cord by regulating oligodendrocytes

In addition to microglia and astrocytes, oligodendrocytes are one of the main glial cell types in the spinal cord. Oligodendrocytes originate from oligodendrocyte progenitor cells (OPC) and constitute approximately 5% to 10% of the glial cells [37]. The primary function of oligodendrocytes is to generate myelin, an extended membrane from the cell that wraps tightly around axons. It is critical for the rapid and efficient conduction of electrical impulses along the axon and for maintaining its integrity [38]. Research has found that Treg cells are strongly involved in oligodendrocyte differentiation and myelination, thus positively affecting the recovery process for SCI [36].

To verify whether Treg cells were functionally important in oligodendrocyte differentiation. Dombrowski et al. [39] found that Treg-depleted animals exhibited significantly less-differentiated CC1^+^Olig2^+^ oligodendrocytes (mature oligodendrocytes). Subsequently, they discovered that Olig2^+^Ki67^+^ proliferating OPC presents no significant differences at 5 days post-lesion or 10 days post-lesion, excluding inadequate recruitment of OPC in the demyelinated region. This research indicates that the decreased generation of oligodendrocytes was probably due to an impairment in OPC differentiation. The administration of Treg cells significantly increased the number of differentiated oligodendrocytes in Treg-deficient animals, indicating the capacity of Treg cells to rescue impaired oligodendrocyte differentiation. They further explored the signalling pathways: Treg cells can produce CCN3 and promote oligodendrocyte differentiation and myelination. CCN3 is a growth regulatory protein, biologically active in the extracellular and nuclear compartments, and involved in tissue regeneration [40]. The production of CCN3 by Treg cells was verified by a double ELISA and a Western blot. To determine whether CCN3 mediates oligodendrocyte differentiation, Dombrowski et al. [39] showed that the anti-CCN3 antibody eliminated Treg cells-induced oligodendrocyte differentiation, and suppressed the pro-myelinating effect of Treg cells in the slice cultures. Furthermore, treatment with the infusion of CCN3 significantly promoted myelination in slices. These studies demonstrated that CCN3, a novel protein secreted by Treg cells, mediated Tregs-driven oligodendrocyte differentiation and myelination.

### Treg cells repair the spinal cord by regulating astrocytes

Astrocytes (also known as glia) are the most abundant resident glial cells in CNS, along with oligodendrocytes and microglia [41]. Under normal physiological conditions, astrocytes typically provide trophic support to neurons and promote synapse formation [42]. When the local immune microenvironment changes after SCI, danger signals induce astrocytes to produce glial fibrillary acidic protein (GFAP). Astrocytes upregulate GFAP in response to most types of CNS injury and are widely used as a marker of astrocyte reactivity [43]. Neuroinflammation and ischemia induced two different subtypes of reactive astrocytes. It might be argued that new names are required to describe the variety of reactive astrocytes adequately; however, the existing information does not yet provide objective categorization of reactive astrocytes. Also, it is advisable to avoid using ambiguous and binary phrases when defining astrocyte phenotypes, since they are too simplistic to be meaningful unless they are supported by precise molecular mechanisms and direct causative experimental data [44]. A1 cells (A1s) induced by activated microglia [42], lose most normal astrocyte functions but acquire a novel neurotoxic activity that rapidly kills neurons and matures differentiated oligodendrocytes. In contrast, A2 cells (A2s) up-regulated many neurotrophic factors, so we postulated that A2s are protective. However, local astrocyte progenitors near the injured tissue develop the glial scar as the inflammatory response progresses. Although the glial scar is thought to be the main barrier to the restoration of neuronal connectivity and axonal regeneration, it initially serves as a barrier by isolating the injured area, avoiding the proliferation of inflammatory cells, establishing an environment that is favorable for surviving neurons surviving neurons, and maintaining the blood–brain barrier (BBB) [45].

Recently, many studies have found that Treg cells can secret amphiregulin (AREG) to promote the recovery of the spinal cord [46]. ARGE, a low-affinity epidermal growth factor receptor (EGFR) ligand, is involved in wound healing and tissue repair [46]. Ito et al. [47] found that Treg cells expressed high levels of AREG after 14 days. The reinfusion of AREG lessened the neurological dysfunction by reducing the expression of neurotoxic astrocyte genes, the number of astroglioses, and the number of apoptotic neurons in Treg-cell-depleted mice. Furthermore, wild-type Treg cells could inhibit excessive astrogliosis, neurological impairments, and the production of neurotoxic genes. These results indicate that AREG from Treg cells regulates astrogliosis and promotes neurological recovery. They found that the IL-6–STAT3 pathway is highly activated by Treg cell depletion. To illustrate the relationship between AREG and the IL-6–STAT3 pathway, they discovered that IL-6 greatly increased the expression of GFAP and STAT3 in primary astrocytes. According to these findings, IL-6–STAT3 pathway modulation by AREG in astrocytes is a crucial step in the neuroprotective role of Treg cells.

It has been mentioned above that astrocytes can be neurotoxic and neuroprotective. However, increasing research demonstrates that several astrocyte-derived factors also exhibit dual characteristics [48, 49]. These findings may involve many molecular mechanisms and microenvironments related to the various subtypes, damage zones, and phases of neurotrauma. Therefore, either inhibiting or promoting reactive astrogliosis has no therapeutic value. A promising astrocyte-targeting therapeutic approach is to selectively stimulate the beneficial astrocyte-derived molecules while attenuating the deleterious ones based on the spatiotemporal environment.

### Treg cells repair the spinal cord by regulating other cells

Mesenchymal stem cells (MSCs) are adult stem cells derived from the mesoderm in early embryonic development with self-renewal, multi-directional differentiation potential. They can maintain their biological characteristics after large-scale expansion in vitro. The therapeutic effects of MSCs are currently considered to have two aspects: one is the directional differentiation and replacement repair of damaged tissue by MSCs; the other is the paracrine effect of MSCs. Bone marrow mesenchymal stem cells (BMSCs) is a well-characterized cell population, consisting of adherent monocytes extracted from bone marrow. Harvested and isolated BMSC populations may contain more mature cell types that protect against harmful inflammation. Research has confirmed that Foxp3, a critical transcription factor responsible for Treg maturation [50], is expressed in mesenchymal stem cells [51]. Neal et al. [52] found a distinct subset of CD4^+^/CD25^+^/Foxp3^+^ Treg cells in BMSCs. Treg cells minimized the production of IL-6 (a pro-inflammatory cytokine) and inhibited BMSC secretion of FGF-b (a cytokine associated with BMSC proliferation and differentiation) [53]. In addition, the proportion of Treg cells naturally found in BMSCs is optimal to provide the most significant neuroprotective benefit of stem cell therapy. Several clinical trials are ongoing up to this point, and MSC-based treatment maybe becomes a common treatment in future [54, 55].

Research has found that Treg cells also affect the blood–spinal cord barrier (BSCB) to promote spinal cord recovery through neutrophils. The destruction of the BSCB is a prerequisite for immune cells to enter the injury site and hinders the prognosis of the secondary injury. Treg cells attenuated barrier disruption following ischemia/reperfusion and subsequent infiltration of peripheral inflammatory cells. Jin et al. [56] describe a neuroprotective mechanism by which Treg cells inhibit neutrophil-derived matrix metalloproteinase 9 (MMP9). MMP9 is a metalloproteinase that can damage the barrier, especially the intercellular matrix. Several related experiments have proven that knocking out the MMP9 gene or applying MMP9 inhibitors can reduce the damage to the barrier after injury.

### The adverse role of Treg cells

While there is plenty of evidence to support its positive effects, there is some evidence for the adverse role of Treg cells after SCI. Walsh et al. [57] found that a small amount of Treg cells was necessary to promote the recovery of neuronal injury. In contrast, a large amount of Treg cells aggravated the injury process. In addition, in vitro-induced Treg cells were infused back into mice to suppress the response of effector T cells. They obtained the same result, namely reducing the number of neurons. Their findings are consistent with the idea that a spontaneous immune response following CNS damage is advantageous and is closely controlled by Treg cells. Removal of Treg causes an excessive immune response that harms injured tissue. However, injecting excessive Treg cells or potentiating their suppressive function hinders a healthy immunological response to injury and impairs neurons' survival.

### The status of treatment and the prospects of cell-based therapies

Neurological pathophysiology is a complex and dynamic set of cellular and molecular events after SCI [58]. Nowadays, the main treatment for SCI includes physical therapy, drug treatment, and surgery [59]. However, the outcome for patients with SCI remains unsatisfactory. For the treatment of SCI, we also must develop new and effective therapeutics. In recent years, cell therapies for SCI have gradually become a new research hotspot [60]. Using Treg cells as a cell-based treatment method was initially proven in mouse models, where Treg cells were found to have a beneficial role in pathogenesis. Transplantation of Treg cells could alleviate autoimmune disease [61, 62]. Numerous phase I and phase II clinical trials utilizing ex vivo expanded Treg cells to treat autoimmune illnesses [63, 64]. Cell therapy trials in this area still face significant obstacles, such as meeting strict regulatory requirements, assuring adequately powered efficacy trials, and securing sustainable long-term funding [65]. Within preclinical research, spinal cord repair techniques are moving away from simple cell-only injections and exploring therapeutic approaches where cells are delivered with biomaterials [66]. Biomaterials may help cells survive and provide essential structural support for both transplanted cells and regenerating host tissue. This gives us a new approach for effectively delivering regulatory T cells. To improve cell delivery, enhance cell survival, scale up tissue engineering technology, and facilitate improved functional recovery, future trials may seek to utilize realistic and scalable tissue engineering technologies [65].

## Conclusion

This review provides an overview of the regulatory role of Treg cells in spinal cord injury. Several mechanisms contribute to the prognosis of spinal cord injury. Future research is anticipated to expand on these first technological achievements and utilize combinations of biomaterials to enhance the Tregs' capability for survival to repair the spinal cord.

## Data Availability

Not applicable.

## References

[1] Nakamura M, Okano H (2013). Cell transplantation therapies for spinal cord injury focusing on induced pluripotent stem cells. Cell Res.

[2] Khorasanizadeh M, Yousefifard M, Eskian M, Lu Y, Chalangari M, Harrop JS (2019). Neurological recovery following traumatic spinal cord injury: a systematic review and meta-analysis. J Neurosurg Spine.

[3] Hutson TH, Di Giovanni S (2019). The translational landscape in spinal cord injury: focus on neuroplasticity and regeneration. Nat Rev Neurol.

[4] Courtine G, Sofroniew MV (2019). Spinal cord repair: advances in biology and technology. Nat Med.

[5] Ahuja CS, Wilson JR, Nori S, Kotter MRN, Druschel C, Curt A (2017). Traumatic spinal cord injury. Nat Rev Dis Primers.

[6] Ahuja CS, Fehlings M (2016). Concise review: bridging the gap: novel neuroregenerative and neuroprotective strategies in spinal cord injury. Stem Cells Transl Med.

[7] Fan B, Wei Z, Feng S (2022). Progression in translational research on spinal cord injury based on microenvironment imbalance. Bone research.

[8] Tran AP, Warren PM, Silver J (2018). The biology of regeneration failure and success after spinal cord injury. Physiol Rev.

[9] Wang PF, Qi XB, Xu GH, Liu JN, Guo JC, Li X (2019). CCL28 promotes locomotor recovery after spinal cord injury via recruiting regulatory T cells. Aging-Us.

[10] Sakaguchi S, Yamaguchi T, Nomura T, Ono M (2008). Regulatory T cells and immune tolerance. Cell.

[11] Sakaguchi S, Miyara M, Costantino CM, Hafler DA (2010). FOXP3+ regulatory T cells in the human immune system. Nat Rev Immunol.

[12] Scheinecker C, Göschl L, Bonelli M (2020). Treg cells in health and autoimmune diseases: new insights from single cell analysis. J Autoimmun.

[13] Hu X, Leak RK, Thomson AW, Yu F, Xia Y, Wechsler LR (2018). Promises and limitations of immune cell-based therapies in neurological disorders. Nat Rev Neurol.

[14] Machhi J, Kevadiya BD, Muhammad IK, Herskovitz J, Olson KE, Mosley RL (2020). Harnessing regulatory T cell neuroprotective activities for treatment of neurodegenerative disorders. Mol Neurodegener.

[15] Milich LM, Ryan CB, Lee JK (2019). The origin, fate, and contribution of macrophages to spinal cord injury pathology. Acta Neuropathol.

[16] Bennett FC, Bennett ML, Yaqoob F, Mulinyawe SB, Grant GA, Hayden Gephart M (2018). A combination of ontogeny and CNS environment establishes microglial identity. Neuron.

[17] Shemer A, Grozovski J, Tay TL, Tao J, Volaski A, Süß P (2018). Engrafted parenchymal brain macrophages differ from microglia in transcriptome, chromatin landscape and response to challenge. Nat Commun.

[18] Orihuela R, McPherson CA, Harry GJ (2016). Microglial M1/M2 polarization and metabolic states. Br J Pharmacol.

[19] Francos-Quijorna I, Sánchez-Petidier M, Burnside ER, Badea SR, Torres-Espin A, Marshall L (2022). Chondroitin sulfate proteoglycans prevent immune cell phenotypic conversion and inflammation resolution via TLR4 in rodent models of spinal cord injury. Nat Commun.

[20] Rong Y, Wang Z, Tang P, Wang J, Ji C, Chang J (2023). Engineered extracellular vesicles for delivery of siRNA promoting targeted repair of traumatic spinal cord injury. Bioact Mater.

[21] Dumas AA, Borst K, Prinz M (2021). Current tools to interrogate microglial biology. Neuron.

[22] Paolicelli RC, Sierra A, Stevens B, Tremblay ME, Aguzzi A, Ajami B (2022). Microglia states and nomenclature: a field at its crossroads. Neuron.

[23] Liu R, Li Y, Wang Z, Chen P, Xie Y, Qu W (2023). Regulatory T cells promote functional recovery after spinal cord injury by alleviating microglia inflammation via STAT3 inhibition. CNS Neurosci Ther.

[24] Cai J, Wang D, Zhang G, Guo X (2019). The role Of PD-1/PD-L1 axis in treg development and function: implications for cancer immunotherapy. Onco Targets Ther.

[25] Gianchecchi E, Fierabracci A (2018). Inhibitory receptors and pathways of lymphocytes: the role of PD-1 in Treg development and their involvement in autoimmunity onset and cancer progression. Front Immunol.

[26] Lasorella S, Porto R, Iezzi ML, Pistone C, Marseglia GL, Verrotti A (2020). Comparison of triptorelin acetate vs triptorelin pamoate in the treatment of central precocious puberty (CPP): a retrospective study. Gynecol Endocrinol.

[27] Yao A, Liu F, Chen K, Tang L, Liu L, Zhang K (2014). Programmed death 1 deficiency induces the polarization of macrophages/microglia to the M1 phenotype after spinal cord injury in mice. Neurotherapeutics.

[28] He X, Lin S, Yang L, Tan P, Ma P, Qiu P (2021). Programmed death protein 1 is essential for maintaining the anti-inflammatory function of infiltrating regulatory T cells in a murine spinal cord injury model. J Neuroimmunol.

[29] Yang Z, Yu A, Liu Y, Shen H, Lin C, Lin L (2014). Regulatory T cells inhibit microglia activation and protect against inflammatory injury in intracerebral hemorrhage. Int Immunopharmacol.

[30] Lech M, Anders HJ (2013). Macrophages and fibrosis: How resident and infiltrating mononuclear phagocytes orchestrate all phases of tissue injury and repair. Biochem Biophys Acta.

[31] Gagliani N, Amezcua Vesely MC, Iseppon A, Brockmann L, Xu H, Palm NW (2015). Th17 cells transdifferentiate into regulatory T cells during resolution of inflammation. Nature.

[32] Kovacs SB, Miao EA (2017). Gasdermins: effectors of pyroptosis. Trends Cell Biol.

[33] Shi J, Gao W, Shao F (2017). Pyroptosis: gasdermin-mediated programmed necrotic cell death. Trends Biochem Sci.

[34] Wang L, Hauenstein AV (2020). The NLRP3 inflammasome: Mechanism of action, role in disease and therapies. Mol Aspects Med.

[35] Chio JCT, Wang J, Badner A, Hong J, Surendran V, Fehlings MG (2019). The effects of human immunoglobulin G on enhancing tissue protection and neurobehavioral recovery after traumatic cervical spinal cord injury are mediated through the neurovascular unit. J Neuroinflammation.

[36] Al Mamun A, Wu Y, Monalisa I, Jia C, Zhou K, Munir F (2021). Role of pyroptosis in spinal cord injury and its therapeutic implications. J Adv Res.

[37] Bradl M, Lassmann H (2010). Oligodendrocytes: biology and pathology. Acta Neuropathol.

[38] Kuhn S, Gritti L, Crooks D, Dombrowski Y (2019). Oligodendrocytes in development, myelin generation and beyond. Cells.

[39] Dombrowski Y, O'Hagan T, Dittmer M, Penalva R, Mayoral SR, Bankhead P (2017). Regulatory T cells promote myelin regeneration in the central nervous system. Nat Neurosci.

[40] Wang X, He H, Wu X, Hu J, Tan Y (2014). Promotion of dentin regeneration via CCN3 modulation on notch and BMP signaling pathways. Biomaterials.

[41] Freeman MR (2010). Specification and morphogenesis of astrocytes. Science (New York, NY).

[42] Giovannoni F, Quintana FJ (2020). The role of astrocytes in CNS inflammation. Trends Immunol.

[43] Sofroniew MV (2014). Astrogliosis. Cold Spring Harb Perspect Biol.

[44] Escartin C, Galea E, Lakatos A, O'Callaghan JP, Petzold GC, Serrano-Pozo A (2021). Reactive astrocyte nomenclature, definitions, and future directions. Nat Neurosci.

[45] Zhou Y, Shao A, Yao Y, Tu S, Deng Y, Zhang J (2020). Dual roles of astrocytes in plasticity and reconstruction after traumatic brain injury. Cell Commun Signal.

[46] Zaiss DMW, Gause WC, Osborne LC, Artis D (2015). Emerging functions of amphiregulin in orchestrating immunity, inflammation, and tissue repair. Immunity.

[47] Ito M, Komai K, Mise-Omata S, Iizuka-Koga M, Noguchi Y, Kondo T (2019). Brain regulatory T cells suppress astrogliosis and potentiate neurological recovery. Nature.

[48] Ding ZB, Song LJ, Wang Q, Kumar G, Yan YQ, Ma CG (2021). Astrocytes: a double-edged sword in neurodegenerative diseases. Neural Regen Res.

[49] Fan YY, Huo J (2021). A1/A2 astrocytes in central nervous system injuries and diseases: angels or devils?. Neurochem Int.

[50] Hori S, Nomura T, Sakaguchi S (2003). Control of regulatory T cell development by the transcription factor Foxp3. Science (New York, NY).

[51] Li P, Gan Y, Sun BL, Zhang F, Lu B, Gao Y (2013). Adoptive regulatory T-cell therapy protects against cerebral ischemia. Ann Neurol.

[52] Neal EG, Acosta SA, Kaneko Y, Ji X, Borlongan CV (2019). Regulatory T-cells within bone marrow-derived stem cells actively confer immunomodulatory and neuroprotective effects against stroke. J Cereb Blood Flow Metab.

[53] Kunath T, Saba-El-Leil MK, Almousailleakh M, Wray J, Meloche S, Smith A (2007). FGF stimulation of the Erk1/2 signalling cascade triggers transition of pluripotent embryonic stem cells from self-renewal to lineage commitment. Development (Cambridge, England).

[54] Kouchakian MR, Baghban N, Moniri SF, Baghban M, Bakhshalizadeh S, Najafzadeh V (2021). The clinical trials of mesenchymal stromal cells therapy. Stem Cells Int.

[55] Zhao Y, Tang F, Xiao Z, Han G, Wang N, Yin N (2017). Clinical study of NeuroRegen scaffold combined with human mesenchymal stem cells for the repair of chronic complete spinal cord injury. Cell Transplant.

[56] Jin LY, Li J, Wang KF, Xia WW, Zhu ZQ, Wang CR (2021). Blood-spinal cord barrier in spinal cord injury: a review. J Neurotrauma.

[57] Walsh JT, Zheng JJ, Smirnov I, Lorenz U, Tung K, Kipnis J (2014). Regulatory T cells in central nervous system injury: a double-edged sword. J Immunol.

[58] Fan B, Wei Z, Yao X, Shi G, Cheng X, Zhou X (2018). Microenvironment imbalance of spinal cord injury. Cell Transplant.

[59] Ahuja CS, Nori S, Tetreault L, Wilson J, Kwon B, Harrop J (2017). Traumatic spinal cord injury-repair and regeneration. Neurosurgery.

[60] Ide C, Kanekiyo K (2016). Points regarding cell transplantation for the treatment of spinal cord injury. Neural Regen Res.

[61] Danikowski KM, Jayaraman S, Prabhakar BS (2017). Regulatory T cells in multiple sclerosis and myasthenia gravis. J Neuroinflammation.

[62] Morgan ME, Flierman R, van Duivenvoorde LM, Witteveen HJ, van Ewijk W, van Laar JM (2005). Effective treatment of collagen-induced arthritis by adoptive transfer of CD25+ regulatory T cells. Arthritis Rheum.

[63] Martelli MF, Di Ianni M, Ruggeri L, Falzetti F, Carotti A, Terenzi A (2014). HLA-haploidentical transplantation with regulatory and conventional T-cell adoptive immunotherapy prevents acute leukemia relapse. Blood.

[64] Marek-Trzonkowska N, Myśliwiec M, Dobyszuk A, Grabowska M, Derkowska I, Juścińska J (2014). Therapy of type 1 diabetes with CD4(+)CD25(high)CD127-regulatory T cells prolongs survival of pancreatic islets—results of one year follow-up. Clin Immunol (Orlando, Fla).

[65] Bartlett RD, Burley S, Ip M, Phillips JB, Choi D (2020). Cell Therapies for spinal cord injury: trends and challenges of current clinical trials. Neurosurgery.

[66] Amer MH, Rose F, Shakesheff KM, Modo M, White LJ (2017). Translational considerations in injectable cell-based therapeutics for neurological applications: concepts, progress and challenges. NPJ Regen Med.

